# Differential Monocyte Actuation in a Three‐Organ Functional Innate Immune System‐on‐a‐Chip

**DOI:** 10.1002/advs.202000323

**Published:** 2020-06-02

**Authors:** Trevor Sasserath, John W. Rumsey, Christopher W. McAleer, Lee Richard Bridges, Christopher J. Long, Daniel Elbrecht, Franz Schuler, Adrian Roth, Christina Bertinetti‐LaPatki, Michael L. Shuler, James J. Hickman

**Affiliations:** ^1^ Hesperos, Inc. 12501 Research Parkway, Suite 100 Orlando FL 32826 USA; ^2^ Hoffmann‐La Roche Pharmaceuticals Division Bldg 73, Rm 117b Basel 4070 Switzerland; ^3^ NanoScience Technology Center, University of Central Florida 12424 Research Parkway, Suite 400 Orlando FL 32826 USA

**Keywords:** animal model alternatives, functional data, human‐on‐a‐chip, immune system‐on‐a‐chip, in vitro platforms, multiorgan systems, system‐on‐a‐chip

## Abstract

A functional, human, multiorgan, pumpless, immune system‐on‐a‐chip featuring recirculating THP‐1 immune cells with cardiomyocytes, skeletal muscle, and liver in separate compartments in a serum‐free medium is developed. This in vitro platform can emulate both a targeted immune response to tissue‐specific damage, and holistic proinflammatory immune response to proinflammatory compound exposure. The targeted response features fluorescently labeled THP‐1 monocytes selectively infiltrating into an amiodarone‐damaged cardiac module and changes in contractile force measurements without immune‐activated damage to the other organ modules. In contrast to the targeted immune response, general proinflammatory treatment of immune human‐on‐a‐chip systems with lipopolysaccharide (LPS) and interferon‐*γ* (IFN‐*γ*) causes nonselective damage to cells in all three‐organ compartments. Biomarker analysis indicates upregulation of the proinflammation cytokines TNF‐*α*, IL‐6, IL‐10, MIP‐1, MCP‐1, and RANTES in response to LPS + IFN‐*γ* treatment indicative of the M1 macrophage phenotype, whereas amiodarone treatment only leads to an increase in the restorative cytokine IL‐6 which is a marker for the M2 phenotype. This system can be used as an alternative to humanized animal models to determine direct immunological effects of biological therapeutics including monoclonal antibodies, vaccines, and gene therapies, and the indirect effects caused by cytokine release from target tissues in response to a drug's pharmacokinetics (PK)/pharmacodynamics (PD) profile.

## Graphical abstract

A functional, human, multiorgan, pumpless, immune system‐on‐a‐chip containing recirculating THP‐1 immune cells with cardiomyocytes, skeletal muscle, and liver compartments with a serum‐free medium is developed. This system emulates both a targeted immune response to tissue‐specific damage and a holistic immune response to proinflammatory compound exposure. These immune responses are reflected in changes to parenchymal cell functionality and cytokine release.

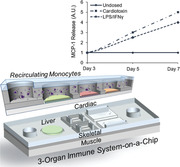

## 1 Introduction

The pharmaceutical industry faces growing challenges in drug discovery and development, especially at the preclinical level for evaluation of small molecules and, in particular, for biologics. Specifically, extended development times combined with low success rates from clinical trials has led to rapidly increasing costs estimated to range from 500 million to over 2 billion dollars for a new therapeutic.^[^ ^1^ ^]^ Furthermore, a recent study indicated that 32% of novel drugs approved by the FDA from 2001 to 2010 were affected by a post‐market safety event indicating a need for more robust efficacy and toxicity data to be generated during the drug development process.^[^ ^2^ ^]^ Evaluation of the body's immune response to a drug at the preclinical stage is especially problematic. This lack of preclinical data to inform clinical trials is relevant for antibody‐based therapy and for other biologics as preclinical trials require humanized animal models, which are expensive. Due to the rising cost of discovery and poor predictability of current models for generating successful drugs, human‐on‐a‐chip (HoaC) or multiorgan, microphysiological systems have seen a growing interest in their use as model systems for drug discovery. Such systems can be used to investigate pharmacodynamics (PD) and pharmacokinetics (PK) of potential drugs with the expectation of reduced cost and increased efficiency in the development process.^[^ ^3^ ^]^

One current limitation of HoaC drug discovery platforms is the absence of a recirculating immune cell to mimic aspects of the immunosurveillance process.^[^ ^4^ ^]^ Absent an immune component, these systems fail to address the potential immune disrupting effects of novel drug compounds in multiorgan systems.^[^ ^5^ ^]^ These effects could range from mild inflammation to extreme cytokine storm induction to immunosuppression. Immune components have been implemented in single organ systems such as resident macrophages in liver,^[^ ^6^ ^]^ lung,^[^ ^7^ ^]^ and others^[^ ^8^, ^9^ ^]^ but have not been demonstrated in recirculating multiorgan systems. An integrated immune component in a HoaC system with a recirculating blood surrogate would facilitate seamless integration of immune‐related information with functional and biomarker readouts for efficacy and toxicity evaluation of novel compounds dosed acutely and chronically. Collecting immune response data in parallel with noninvasive functional and biomarker data significantly enhances the horizontal scalability of HoaC systems enhancing their application for both broad‐based and targeted drug discovery programs.

The immune system coordinates with other organ systems to orchestrate the activities necessary to combat infection, eliminate damaged cells, repair tissue and return to basal physiology, thereby maintaining homeostasis. One important immune system cell type for both cytokine production and cell–cell interactions are monocytes. Monocytes are ubiquitous sentry immune cells involved in wound healing, pathogen elimination, and activating the adaptive immune response, to facilitate two general immune response paradigms. Proinflammatory stimulation by molecules such as TNF‐*α*, lipopolysaccharide (LPS), and interferon‐*γ* (IFN‐*γ*) leads to a classical activation of monocytes to macrophages resulting in tissue infiltration and pathogen and/or tumor cell elimination that can be tissue specific or systemic. In addition, an immunoregulating, tissue remodeling phenotype driven largely by damaged tissues, and stimulated by IL‐6 and IL‐10, is also present.^[^ ^10^, ^11^ ^]^ For example, patients receiving the antiarrhythmic drug, amiodarone, showed monocyte/macrophage infiltration into tissues damaged by the treatment.^[^ ^12^ ^]^ In both cases, circulating monocytes were activated by cytokines and other signaling molecules, extravasated from the bloodstream, migrated to sites of infection, or tissue damage and began the process of pathogen removal or tissue repair.^[^ ^13^ ^]^ While the role for cytokines in response to site‐directed as opposed to holistic proinflammation is complex, it is generally accepted that self‐resolving inflammation involving IL‐6 and TNF‐*α* is considered reparative while continued elevation of proinflammatory cytokines including TNF‐*α* and RANTES is considered pathological.^[^ ^10^, ^14^ ^]^

Primary activation of macrophages in response to cytokines or drug compounds, or secondarily because of molecules produced by tissues affected by the circulating compound, will result in separate immune responses. Monocyte activation to macrophages and their subsequent interaction with target tissues will vary according to the activation program and tissue damaged. For example, classical (M1) activation, readying the macrophages to eliminate pathogens, will produce a cytokine profile that enhances the immune system's capabilities for pathogen removal and chemotaxis of leukocytes to sites of infection, whereas an alternative phenotype (M2a, 2b, and 2c) will produce a different cytokine profile, and chemotaxis of macrophages to sites of tissue damage.^[^ ^15^ ^]^

In the context of basic and translational research, THP‐1 monocytes have been well characterized. THP‐1 cells have been shown to express CD14 and CD11b after stimulation with phorbol 12‐myristate 13‐acetate (PMA).^[^ ^16^ ^]^ Additionally, THP‐1 cells are capable of polarizing to the M1 or M2 phenotype depending on compound treatment.^[^ ^17^ ^]^ Further, THP‐1 cells in both monocyte and macrophage states have been validated for drug‐screening purposes: compounds include mebendazole, terbinafine, troglitazone, and others.^[^ ^18^, ^19^, ^20^ ^]^ In work conducted by Groger et al., THP‐1 monocytes were cultured with liver organoids in a microphysiological system to study sepsis.^[^ ^21^ ^]^ They observed liver infiltration and monocyte differentiation in response to LPS treatment. The use of these cells is more advantageous than peripheral blood mononuclear cell (PBMC)‐derived monocytes due to their availability, ease of culture, purity, homogenous genetic background, and inactivated baseline status.^[^ ^22^ ^]^ An important limitation of any cell line, including THP‐1 cells, is the possibility of nonphysiological responses by the cells when cultured in controlled conditions.^[^ ^23^ ^]^ However, this limitation may be overcome by providing the relevant interactions with somatic cells present in their natural environment, something a multiorgan system provides.^[^ ^24^ ^]^

In previous studies, we demonstrated the effects of drugs dosed in a four‐organ system composed of liver, skeletal muscle, cardiomyocytes, and neurons, as well as in a 2‐organ heart liver system that established the ability of in vitro PKPD models to predict in vivo results.^[^ ^25^, ^26^, ^27^ ^]^ We also evaluated concurrently the efficacy and off‐target toxicity of drugs and their metabolites in a multiorgan cancer model.^[^ ^28^ ^]^ Another manuscript showed the ability of a 4‐organ system, composed of heart, liver, skeletal muscle, and neurons, to remain functionally competent for 28 days in a recirculating serum‐free medium.^[^ ^29^ ^]^ Each microphysiological (MPS) system was characterized and designed utilizing computational fluid dynamic modeling (CFD) to calculate shear stress to be below 0.05 dynes cm^−2^ at the bottom of each chamber where the cells were located.^[^ ^29^ ^]^ Taken together, these studies demonstrate the effectiveness of microphysiological, human‐on‐a‐chip systems to investigate immune cell–parenchymal cell interactions in response to distinct compound treatment strategies in a validated platform.

Here, we demonstrate a multiorgan human model integrated with an immune component represented by monocytes capable of responding to either indirect activation resulting from cytokines produced by damaged tissue or broad activation that is a direct response to signaling molecules. These different approaches to monocyte‐macrophage activation led to distinctly different responses by the system in terms of cytokine production and cell‐cell interactions while also facilitating interrogation of affected organs in the recirculating system using functional and biomarker readouts. This HoaC immune system model could find application in drug discovery for systemic diseases, including cancer and inflammatory disorders such as sepsis, where the complex interaction of drugs, immune system and other organ systems is of paramount importance.^[^ ^30^ ^]^ This is especially important for biologics utilized in oncology where animal models do not represent these adverse human interactions reproducibly.^[^ ^31^ ^]^ Furthermore, the system has targeted applications for improving drug discovery for autoimmune diseases and to enhance studies of autoimmune disease treatment for precision medicine.^[^ ^32^ ^]^ Moreover, the ability of this HoaC model to recreate a subset of the diverse and dynamic interactions between immune cells and their organ system counterparts and the responses of those cells to novel drug compounds increases the sophistication of HoaC systems and brings patient healthcare one step closer to realizing personalized medicine.

## 2 Experimental Section

### 2.1 System Configuration of the Three‐Organ System with and Without Immune Component

The 3‐O system was designed to contain a liver module and biological microelectromechanical systems (BioMEMS) devices to noninvasively measure cardiomyocytes’ electrical (MEA) and mechanical (cantilevers) function, skeletal muscle mechanical function (cantilevers), and THP‐1 monocytes suspended in recirculating serum‐free medium (blood surrogate) (Figure S1, Supporting Information). The liver module's activity was monitored in the system using biomarker quantification. 3‐O also served as a THP‐1 cell‐free controls for the 3‐O systems with THP‐1 cells. These systems contained batch‐matched liver and BioMEMs modules without recirculating THP‐1 cells. Cardiac and skeletal muscle physiology was evaluated in part by seeding the cells onto separate custom arrays of microcantilevers. Contractile force of the cardiac and skeletal muscle was calculated from laser‐deflection measurements from cantilever bending resulting from contractile muscle.^[^ ^33^ ^]^ Additionally, cardiomyocyte electrical function was measured from cells cultured on a patterned MEA with a defined conduction path using an MEA amplifier system.^[^ ^33^, ^34^ ^]^ Electrical stimulation for skeletal muscle force was generated inside the system with housing‐embedded electrodes for cantilevers. The long‐term, functional characterization of the organ modules described in this system was previously described.^[^ ^29^ ^]^

In the 3‐O + THP‐1 configuration, each compound's effects were evaluated on liver, cardiac and skeletal muscle function, and immune response in a single device. The systems were operated for 7 days with THP‐1 cell addition on day 0 (day of system assembly), and administration of drugs as a single dose at day 3. Details on the cell culture conditions, material preparation, BioMEMS fabrication and design, and measurements are described in more detail separately.

Convective transport of multiple drug compounds was previously modeled and measured through systems with similar flow profiles,^[^ ^25^, ^27^ ^]^ and while THP‐1 cells are larger and have less diffusivity than small molecules, the transport of both the THP‐1 cells and drug compounds added to this system would be dominated by convective flow. Approximately two times the volume of each chamber was determined to flow through the system in each oscillation in each direction, moving the medium, THP‐1 cells, and cytokines throughout the system. The THP‐1 cells were introduced on one side of the system and were observed to be evenly distributed throughout the system within 24 h. As the transport was dominated by convection (instead of diffusion), this demonstrated effective communication throughout the system via cellular proximity to the recirculating monocytes and exposure to secreted cytokines.

### 2.2 System Fabrication and Assembly

The housing platform was designed using CFDAce^+^ software with compartments capable of holding multiple organ tissue modules connected via microchannels to facilitate medium recirculation. The design also incorporated connections for integrated measurements of mechanical and electrical function of BioMEMS devices. This design was adapted from a similar design for a system accommodating up to 5 organs without a recirculating immune component.^[^ ^26^, ^29^ ^]^ Specifically, the system footprint was minimized, volumes were reduced, and capabilities to record functional measurements were added. The design of the flow chambers and microfluidic device was aided by computational fluid dynamics modeling to establish shear stresses near the cell layers within physiological ranges. This system uses a VWR Signature rocking platform shaker to produce a defined rocking profile of 1 oscillation min^−1^ at an amplitude of 1°. This rocking action produces recirculating flow in the system, with medium flowing between the two reservoirs through the organ chambers. The flow parameters include tilt angle, oscillation rate, dimensions of the channels and chambers, and permissible levels of shear stress. Issues such as bubble formation and adhesion of cells to the surfaces of the system were avoided through bovine serum albumin (BSA) passivation of the systems prior to their assembly. Housings were fabricated from 6 mm thick clear cast acrylic sheets (McMaster‐Carr), and gaskets were made from 0.5 mm thick poly(dimethyl siloxane) (PDMS) elastomer sheet material (Grace BioLabs). Following CFD modeling, 2D drawings were created in Autodesk Inventor to define the laser cutting paths for creating the acrylic housing tops and bottoms as well as the two PDMS gaskets: a bottom gasket for defining the location of each organ chip, and a top gasket to define the fluidic flow paths and fluidic chambers above the chips. Housings and gaskets were laser cut on a Universal Laser Systems Versalaser PLS 75W laser cutter, with additional post processing performed for counterboring screw holes and insertion of brass screw inserts (McMaster‐Carr, 93465A107) for uniform contact pressure distribution throughout the system. The rationale for choice of materials, design of the microfluidic platform, and characterization of flow and shear stress has been previously described.^[^ ^29^ ^]^

Systems were prepared for assembly by dipping the PDMS gaskets in 70% isopropanol, aligning them on the bottom and top acrylic housings, and letting them dry for a minimum of 2 h. When dry, the aligned gaskets and housings were passivated overnight with a solution of 1× phosphate buffered saline (PBS) with 30 mg mL^−1^ bovine serum albumin. The passivation solution was aspirated prior to assembly, a bubble of MOSM was pipetted into the chambers of the bottom housing, and the individual coverslips and BioMEMS devices were removed from their original culture medium and transferred to their respective compartments in the coculture system. Each housing was subsequently covered with the top housing and screwed together using a torque screwdriver set to 40 in‐oz. Assembled housings were placed on rocker platforms in a humidified 37 °C incubator to generate physiological gravity‐driven flow.

### 2.3 Patterned Custom Microelectrode Array (cMEA) and Cantilever Preparation

Cantilever arrays were fabricated following protocols previously described.^[^ ^33^, ^35^, ^36^, ^37^, ^38^, ^39^ ^]^ Briefly, cantilever chips containing cantilevers 4 µm thick, 150 µm wide, and 737 µm long were fabricated from silicon‐on‐insulator wafers with a 4 µm device layer and 1 µm buried oxide. The cantilevers were created in the device layer and a window was produced underneath the cantilevers using standard photolithographic patterning techniques and deep reactive ion etching (DRIE).

Patterned custom microelectrode arrays (cMEAs) were designed with ten 200 µm diameter recording/stimulating electrodes and a single 2000 µm diameter ground electrode and fabricated with standard microfabrication procedures. The wires and electrodes were composed of electron beam evaporated 10 nm titanium and 50 nm platinum and deposited on a fused silica substrate and patterned via a liftoff process. The titanium/platinum wires were insulated with a three‐layer stack of 150 nm layers of silicon oxide, silicon nitride, and silicon oxide produced via plasma‐enhanced chemical vapor deposition (PECVD) and etched using reactive ion etching (RIE) with gas mixtures of CHF_3_ and O_2_.

After fabrication, the surfaces of the cantilever arrays and cMEAs were surface modified using silane chemistry as previously described.^[^ ^34^, ^37^, ^40^ ^]^ The surfaces of the cantilever arrays were modified with 3‐(trimethoxysilyl)propyl)diethylenetriamine silane (DETA) in toluene to produce an amine‐containing surface. Subsequently, fibronectin was adsorbed onto the cantilevers. The surfaces of the cMEAs were patterned with a combination of poly(ethylene glycol) (PEG)‐containing silane and fibronectin. In this method, the surfaces were modified with 2‐[methoxypoly(ethyleneoxy)propyl]trimethoxysilane, a PEG‐containing silane in distilled toluene to produce a cytophobic surface. The PEG monolayer was patterned by firing a 193 nm ArF excimer laser (Lambda Physik, Santa Clara, CA) through a quartz photomask to ablate areas of the PEG chemistry permitting the adsorption of fibronectin as described in the cell culture procedures.

### 2.4 Cell Culture

#### 2.4.1 Primary Human Hepatocyte Culture

Primary human hepatocytes were obtained from single‐patient biopsies through Massachusetts General Hospital (MGH # Lot Hw 054), and cultured in vendor‐specific medium on type I, rat tail collagen‐coated surfaces (60 µg mL^−1^) according to previously published protocols.^[^ ^26^ ^]^ Liver coverslips were monitored for CYP 1A1, 3A4, and 2C9 activity 4 days after drug addition to the systems and production of albumin and urea.

#### 2.4.2 Human Cardiomyocyte Culture

Patterned cMEAs fabricated and cantilevers were coated with human plasma fibronectin (Sigma # FC010‐1MG) diluted in 1× PBS (Thermo Scientific) to concentrations of 10 µg mL^−1^ for cMEAs and 50 µg mL^−1^ for cantilevers, then incubated at 37 °C for 30 min. cMEAs were then washed once and cantilevers washed three times with 1× PBS. Induced pluripotent stem cell (iPSC)‐derived cardiomyocytes (Cellular Dynamics # CMC‐100‐010‐001) were thawed and seeded directly onto the surfaces at 50000 cells per cMEA and 500 000 cells per cantilever array.^[^ ^26^ ^]^

#### 2.4.3 Human Skeletal Muscle Culture

Human skeletal muscle myoblasts, isolated from adult arm or leg muscle, were purchased from Lonza (Lonza CC‐2561). These cells were sourced from a single donor. Cells were expanded through one passage (P1) and cryopreserved. P1 myoblasts were seeded at 500 cells mm^−2^ on patterned cantilevers and grown to confluency (24–48 h) in a custom serum‐free myoblast proliferation medium.^[^ ^41^ ^]^ When confluent, this medium was replaced with a commercial neuronal base medium, NBActiv4 (Brain Bits LLC #Nb4‐500) to induce myoblast fusion into myotubes. The cultures were maintained in NBActiv4 for 11 days before being assembled in the systems.

#### 2.4.4 Human THP‐1 Cell Culture

THP‐1 monocytes (ATCC, TIB‐202) were cultured in T‐75 flasks according to the vendor recommended protocol. Cell culture density was maintained in the range optimal for logarithmic growth (350 000–500 000 cells mL^−1^). THP‐1 cells are prone to forming large aggregates of loosely joined cells. Aggregate formation was discouraged by adding 0.1 mg mL^−1^ of deoxyribonuclease 1 (DNase 1, dissolved in 0.85% NaCl in water) (Sigma‐Aldrich, D4513). When cells had reached their optimal density of 500 000 cells mL^−1^, they were harvested and pelleted via centrifugation at 150 × *g* for 5 min, resuspended in MOSM as previously described, and added to the systems at a physiologically relevant concentration of 250 000 cells mL^−1^.^[^ ^26^ ^]^ THP‐1 cell adhesion to the inside surfaces of the housings was minimized by passivating the inside surfaces with BSA (BSA, 0.5 mg mL^−1^ dissolved in PBS). 10 µL of medium was removed from each system daily to determine the number of freely recirculating cells present. THP‐1 cultures were maintained in RPMI 1640 (ThermoFisher #11875135) with 10% fetal bovine serum (ThermoFisher #16000044) and 1× GlutaMAX (ThermoFisher #35050061) before experimental utilization.

### 2.5 Phase Contrast Cell Imaging

Hepatocyte, cardiomyocyte, skeletal muscle myotube, and THP‐1 cell morphology was documented via phase microscopy on days 3, 5, and 7 after assembly using an inverted phase contrast microscope (Nikon, Eclipse TS100). Images were collected with a stand‐alone microscope camera controller (Nikon, DS‐L3).

### 2.6 Immunocytochemistry

At the end of each experiment, cardiac cMEAs and hepatocyte coverslips were removed from the housings and fixed with 4% paraformaldehyde in 1× PBS for 5 min. Cells were rinsed twice with 1× PBS and permeabilized with a solution of 0.1% Triton X‐100 in 1× PBS for 15 min then blocked with 5% donkey serum in 1× PBS for 30 min. Cells were then incubated overnight at 4 °C with primary antibody in blocking solution. Primary antibodies used were anti‐CD14, anti‐CD16, anti‐CD69, anti‐CD11b, anti‐CD86, and anti‐CCR5. Following three, 5 min washes with 1× PBS, coverslips and cMEAs were incubated for 2 h at room temperature in the dark with a secondary antibody conjugated to Alexa‐488 fluorophore, or Alexa‐568 fluorophore. Following the secondary antibody incubation, surfaces were washed 3× in PBS and incubated with 3 × 10^−3^ m 4ʹ‐6‐diamidino‐2‐phenylindole (DAPI) in 1× PBS for 10 min, in the dark at room temperature for nuclei staining. DAPI solution was removed and the cells were washed 3× with 1× PBS. Finally, coverslips were mounted on glass slides using a Hard‐Set Mounting with DAPI (Vector Labs H‐1400). Alternatively, MEAs were left in the final 1× PBS rinse and imaged using a water immersion lens.

Nonadherent THP‐1 cells were fixed by adding 4% formaldehyde in 1× PBS to the culture medium and incubating at room temperature for 20 min. Cells were pelleted, rinsed once with DI water, and resuspended in 200 µL of DI water. 5 µL of the cell suspension was transferred to the center of a collagen‐coated coverslip and gently smeared using a micropipette tip bent at 90°. Cells were allowed to dry at room temperature for 12 h, then stored at 4°C until imaging. THP‐1 cells were immunostained for CD14, CD16, and CD69.

Fluorescence images were collected using 10× or 40× objectives and 10× magnification of an Axioskop 2 mot plus upright spinning disk confocal microscope (Carl Zeiss), connected to XCite 120 fluorescence illumination system (EXFO) beam, a multispectral laser scanning and Velocity software (Perkin Elmer).

### 2.7 Cell Tracker Studies

THP‐1 cells were collected in a 15 mL conical tube and centrifuged to a pellet at 300 × *g* for 5 min. The cells were resuspended, counted, and the volume adjusted to 1 × 10^6^ cells mL^−1^. The cells were then incubated with Cell Tracker Red CMTPX Dye (ThermoFisher C34552) following the manufacturer's protocol. The dye loaded cells were then used for infiltration studies in 3‐O + THP‐1 systems as described.

### 2.8 Cytochrome p450 1A1 and 3A4 Enzymatic Activity

Cytochrome p450 (Cyp) 1A1, 2C9, and 3A4 activities in hepatocyte cultures were assessed using Promega P450‐Glo Assay kits. Each kit provides a proluciferin substrate probe that gets converted to luciferin once metabolized by its specific target isozyme.^[^ ^26^ ^]^ At coculture endpoint, cells were rinsed 3 times with 1× PBS and incubated with the 1A1 specific substrate, Luciferin‐CEE (120 × 10^−6^ m) (Promega, V8752) in phenol red‐free 1× Dulbecco's modified Eagle medium (DMEM) (Thermo Fisher Scientific, A1443001) for 3 h at 37 °C, at which point samples were collected and stored at −80 °C until further analysis. Similar methods followed for the 2C9 specific substrate, Luciferin‐H (400 × 10^−6^ m) (Promega, V8792), for which incubation time was also 3 h, and the 3A4 specific substrate, Luciferin‐IPA (12 × 10^−6^ m) (Promega, V9002), for which incubation time was 1 h. The cells were washed once with 1× PBS between assays.

Absolute quantification of Cyp activity was performed by further converting the luciferin collected in samples to d‐luciferin, a luminescent compound, then plotting each sample's measured average relative light units (RLUs) on a curve of similarly treated standards ranging from 1 × 10^−9^ to 100 × 10^−9^ m. The conversion reaction involved combining 50 µL of sample or standard with 50 µL of the appropriate luciferin detection reagent (included in Promega P450‐GloAssay kits) in a white opaque 96‐well plate (Cellstar, 655083) and incubating for 20 min in the dark at room temperature. RLUs were measured using a Synergy HT plate reader with KC4 software. The software was programmed to automatically gain adjust the RLU signals of each well and to scale them to the signals of the 100 × 10^−9^ m standards, which were assigned a value of 80 000 RLUs. 3A4, 2C9, and 1A1 samples were measured on separate plates due to large differences in luminescence emission. Each plate was measured a minimum of 5 times to ensure the photomultiplier detector was stabilized, and the average of RLUs was used for numerical analysis. Output values were converted from nm D‐LUC to pmol D‐LUC/h/10^6^ cells.

### 2.9 Drug Preparation and Addition to Systems

Amiodarone hydrochloride (Sigma‐Aldrich, A8423,) was dissolved in dimethyl sulfoxide (DMSO) at a stock concentration of 40 × 10^−3^ m and administered at a final dose of 50 × 10^−6^ m. Lipopolysaccharide (LPS, Sigma‐Aldrich, L2630) was dissolved in PBS 1× at a concentration of 1 µg mL^−1^ and IFN‐*γ* (Sigma‐Aldrich, 11040596001) was dissolved in DI water at a concentration of 100 000 units mL^−1^. LPS and IFN‐*γ* were administered as a single cocktail at final concentrations of 10 ng mL^−1^ and 100 units mL^−1^, respectively. Drug addition to the systems was performed on day 3 post‐assembly. On the day of addition, 3× concentrations of each drug in MOSM were prepared in advance. 1/3 of the medium in each system was removed and replaced with an equal volume of the concentrated drug solution. This facilitated rapid mixing and complete diffusion of the drugs in the systems without requiring a full media change. Medium for analysis of cytokine release was drawn from systems at days 1, 3 (6 h post‐dose), 5, and 7 and was stored at −20 °C until cytokine analysis using a Luminex MAGPIX.

### 2.10 High Pressure Liquid Chromatography (HPLC) and Mass Spectroscopy (MS)

Medium samples collected from the systems were spiked with a tamoxifen internal standard (10 × 10^−9^ m final measured concentration) and combined with acetonitrile at a 1:3 ratio to extract proteins. The mixtures were then centrifuged at 100 × *g* for 5 min and supernatants were collected and diluted 1–51 in the initial mobile phase. A gradient elution method was run for 5.5 min beginning at a ratio of 60:40 and ending at 5:95 of 0.1% formic acid in water:0.1% formic acid in acetonitrile through a 4.6 mm ID × 100 mm, 3.5 × 10^−6^ m Agilent Zorbax C18 column installed in a 1260 Infinity Agilent LC system with a 6490 triple quadrupole MS detector (Agilent). Monitored amiodarone transitions were 646.03 → 58.2 with a 48 eV collision energy for the quantification ion and 646.03 → 100.2 with a 36 eV collision energy for the qualification ion. Quantification and qualification ions for tamoxifen were 372.2 → 72.1 (28 eV CE) and 372.2 → 44 (48 eV), respectively. A calibration curve for amiodarone was prepared using standards ranging from 0.5 × 10^−9^ to 500 × 10^−9^ m. Regression lines were linear with a 1/*x* weighting applied.

### 2.11 Cardiac and Skeletal Muscle Force Measurements

Cardiac and skeletal muscle contractions were measured using a cantilever deflection system as described previously.^[^ ^26^, ^37^, ^38^, ^39^ ^]^ In summary, a helium–neon laser beam was directed from the bottom to the tip of each cantilever and reflected onto a 2D Lateral Effect Position Sensor (Thorlabs, Newton, NJ). Longitudinal deflection of the cantilever due to muscle contractions results in changes in position of the reflected beam on the lateral effect sensor. Contraction strength was analyzed via a manually directed peak detection program written in Python 2.7 that measures the deflection and computes the average maximum peak amplitude and contraction frequency for each cantilever recording. The voltage output from the position sensor was converted directly to force using a modified form of Stoney's equation, as previously published.^[^ ^26^, ^35^, ^36^, ^42^ ^]^

### 2.12 Cardiac Electrical Activity Measurements

Patterned MEAs were fabricated to allow for simultaneous stimulation and recording of cardiomyocyte syncytium electrical activity (100 µm diameter electrodes, 1000 µm between electrodes). Before cell culture, the surfaces were modified to promote cell adhesion in an unbroken “U” pattern that connected multiple electrodes, allowing conduction velocity of the cells to be recorded at different points (14 mm total path length). Patterned cMEAs were introduced to the housings during system assembly on day 0 of the experiment.^[^ ^33^ ^]^

Printed circuit boards (PCBs) and flexible elastomeric connectors (zebra connectors, FUJIPOLY, 1 mm wide × 18.2 mm long × 9 mm tall) were incorporated into each system to create an interface between the cMEA and a commercially available 60 electrode amplifier (MEA1060, Multichannel‐systems). This amplifier was used to both record spontaneous and stimulated electrical activity of the cardiomyocytes. A stimulus generator (STG 1002, Multichannel Systems) was used to stimulate the cells (800 mV rectangular pulse, 0.5–3.0 Hz in 0.25 Hz increments). The multichannel systems software suite was used to control the amplifier and stimulator, and to record action potential traces.

Drug additions were performed on day 3 of the experiment immediately following baseline measurements of spontaneous and stimulated cell activity. Cell activity was recorded again at 48 and 96 h after drug exposure. Conduction velocity (CV), spontaneous beat frequency, and minimum interspike interval (MISI) were extracted from the data using Clampfit (Axon Instruments).

Spontaneous beat frequency was obtained from recordings of cardiac activity 20 s in length. For each recording, the frequency was calculated as the number of measured action potentials divided by the length of time between the first and last action potential during the recording interval. The number of action potentials used in the calculation was decreased by 1 to account for the distances between beats

(1) $$\begin{array}{ccc} \mathrm{BF}\left(\mathrm{Hz}\right) & = & \left(\#\;\mathrm{of}\;\mathrm{peaks}\;\mathrm{in}\;\mathrm{interval}-1\right)/\\ & & \mathrm{time}\;\mathrm{between}\;\mathrm{first}\;\mathrm{and}\;\mathrm{last}\;\mathrm{peaks}\;\left(s\right) \end{array}$$

Conduction velocity was measured by determining the length of time for an action potential to travel from an electrically stimulated electrode along the patterned cardiomyocyte path to various electrodes at specific distances along the path, following the method described in Stancescu et al.^[^ ^33^ ^]^ Stimulation of cardiomyocytes on a single electrode along with the patterned syncytium allowed for a defined conduction path of aligned cardiomyocytes. From the recording of stimulated conduction, conduction velocity was determined by averaging several measurements of the length of time for the action potential to travel along the defined path between two defined electrodes and dividing the physical distance between those two electrodes by that average propagation time

(2) $$\begin{array}{ccc} \mathrm{CV}\left(m\;s^{-1}\right) & = & \mathrm{distance}\;\mathrm{between}\;\mathrm{electrodes}\left(m\right)/\\ & & \mathrm{average}\;\mathrm{signal}\;\mathrm{propagation}\;\mathrm{time}\left(s\right) \end{array}$$

### 2.13 Urea and Albumin Quantification

Human serum albumin (HSA) and urea production was quantified by following previously described protocols.^[^ ^26^ ^]^ Briefly, aliquots of medium drawn from the systems on days 1, 3, 5, and 7 were stored frozen at −20 °C. Samples were then run through commercially available ELISA kits to detect HSA (Bethyl Laboratories Inc, Cat #: E101) and urea (QuantiChrom Urea Assay Kit) (BioAssay Systems, Cat #: DIUR‐500). Data was collected using a BioTek Synergy HT plate reader for absorbance read at 620 nm.

### 2.14 Cell Viability

Hepatocytes coverslips were removed from the systems on the day of disassembly and their viability was determined via a standard MTT assay.^[^ ^26^ ^]^ Briefly, MTT powder was dissolved in a common growth medium to a final concentration of 5 mg mL^−1^. Cells were incubated in 500 µL of the solution for 90 min at 37 °C, 5% CO_2_. The medium was removed, and resultant crystals were dissolved in a lysis buffer consisting of 10% SDS with 0.5% acetic acid in (DMSO. 100 µL of the solution was placed into a 96 well plate and absorbance read at 570 nm using a BioTek Synergy HT plate reader. The viability of cardiac and skeletal muscle cells plated on the BioMEMS devices was assessed using an alamar blue solution (Thermo Fisher, DAL1025). Surfaces were incubated in 500 µL of a 10% solution (v:v) of alamar blue in growth medium at 37 °C, 5% CO_2_ for 24 h. 100 µL of the solution was placed into a 96 well plate and read at fluorescence excitation wavelength 570 nm and emission at 590 nm using the BioTeK Synergy HT plate reader. In order to optimize workflow, the MTT assay was used for hepatocytes and alamar blue for the neuronal, skeletal muscle, and cardiac modules.

### 2.15 Statistical Methods

Values are expressed as the mean ± standard error of the mean (SEM) of a minimum of three independent experiments. Data points were collected such that significant differences were able to be observed in the data, *N* = 3–8 depending on the condition being presented. Data was evaluated with unpaired Student's *t*‐test when analyzing the overall change between two conditions or drug treatment and control. Student's *t*‐test analysis was run with a two‐tail distribution and homo‐ or heteroscedastic variances. For statistical analysis of cytokine release profiles, comparisons were drawn between 3‐O and 3‐O + THP‐1 systems, then between 3‐O + THP‐1 and 3‐O + THP‐1 + amiodarone or 3‐O + THP‐1 + IFN‐*γ*/LPS systems. A one‐way ANOVA test was used to study differences in functional readouts between conditions at each time point (day). Both analyses were run with SigmaPlot and asterisks were used to indicate ranges of *p* values (*p ≤ 0.05, **p ≤ 0.01, ***p ≤ 0.001).

## 3 Results

### 3.1 System Design and Modeling

**Figure 1:**
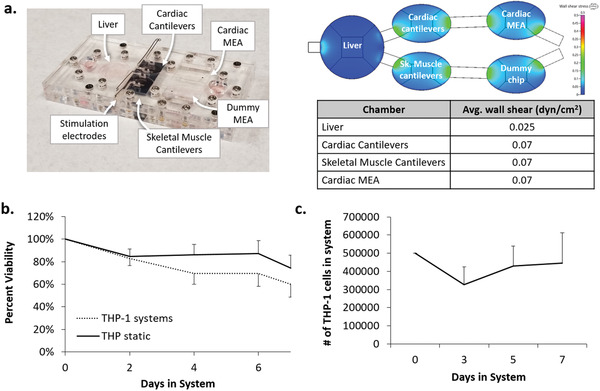
Recirculating THP‐1 cells in four‐organ Systems. a) photograph of the multiorgan housing system with a corresponding color‐coded shear stress plot. A sinusoidal rocking profile was utilized, resulting in a range of shear stresses in each compartment. Given wall shear stress values of chambers containing bioMEMs devices are calculated to fluctuate about an average of ≈0.05 dynes cm^−2^, b) percentage of live THP‐1 cells in systems under gravity‐driven flow versus static cultures throughout the seven‐day experiment, and c) total number of recirculating cells during the seven‐day experiment.

A five‐compartment microfluidic device was developed for this project (**Figure** **1**a) using a previously described multiorgan microfluidics platform as a framework^[^ ^29^ ^]^ (Figure S1, Supporting Information). The flow of medium through the system was gravity‐driven, generated using continuous sinusoidal rocking on a rocking platform. CFD modeling was used to define which parameters needed to be altered to effectively model physiological flow and shear stress in the system, while maintaining an optimal amount of fluid mixing according to physiological parameters.^[^ ^43^, ^44^ ^]^ Shear stress and flow rates were adjusted in vitro by varying the tilt angle and oscillation frequency to match the determined parameters.

### 3.2 Characterization of the Unstimulated 3‐Organ + THP‐1 Immune Body‐On‐A‐Chip System

THP‐1 cells, a monocyte/macrophage cell line, were selected as the immune cell component because they play an important role in immunomodulation, pathogen clearance, wound repair and tissue remodeling after injury.^[^ ^22^ ^]^ After their addition to the assembled systems, a stable population of viable THP‐1 cells, similar to cells in static wells, was maintained in the platform for up to seven days post‐addition (Figure 1b). The THP‐1 cells maintained their ability to proliferate, allowing day‐to‐day counts of recirculating cells to stay constant despite their partial removal from the systems during the daily 30% medium changes (Figure 1b). THP‐1 cells were observed to be recirculating freely for seven days in control systems and exhibited minimal adhesion to the materials in the system (Videos S1–S4 in the Supporting Information indicating recirculation on days 1, 3, 5, and 7).

**Figure 2:**
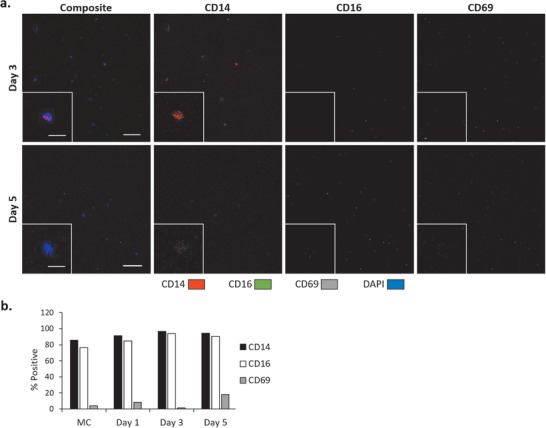
Recirculating THP‐1 cell characterization. a) Expression of CD14, CD16, and CD69 by THP‐1 cells at days 3 and 5 as evaluated by immunocytochemistry, b) flow cytometry quantification of CD expression by THP‐1 cells evaluated at days 1, 3, and 5 in recirculation compared to static monocultures (MC) at day 5. Scale bars = 100 µm in low magnification images, 25 µm in the higher magnification inserts.

**Figure 3:**
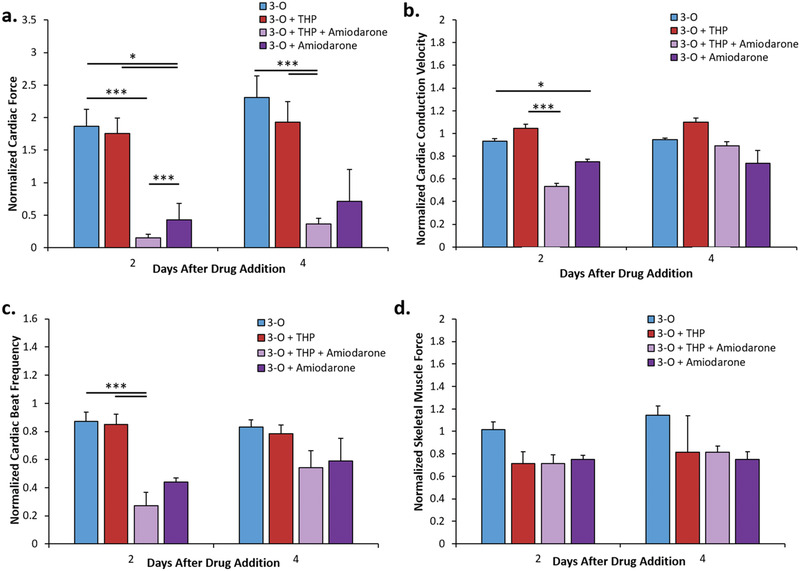
Functional evaluation of cardiomyocytes and skeletal muscle myotubes in amiodarone treated 3‐O + THP‐1 systems. a) Normalized cardiac force measurements from cantilevers, b) normalized cardiac conduction velocity measurements from MEAs, c) normalized beat frequency measurements from cantilevers, and d) normalized skeletal muscle force measurements from cantilevers. Data points are mean ± SEM, *p ≤ 0.05, **p ≤ 0.01, ***p ≤ 0.001. No significant differences observed in panel (b) day 4, panel (d) day 2, and panel (d) day 4.

**Figure 4:**
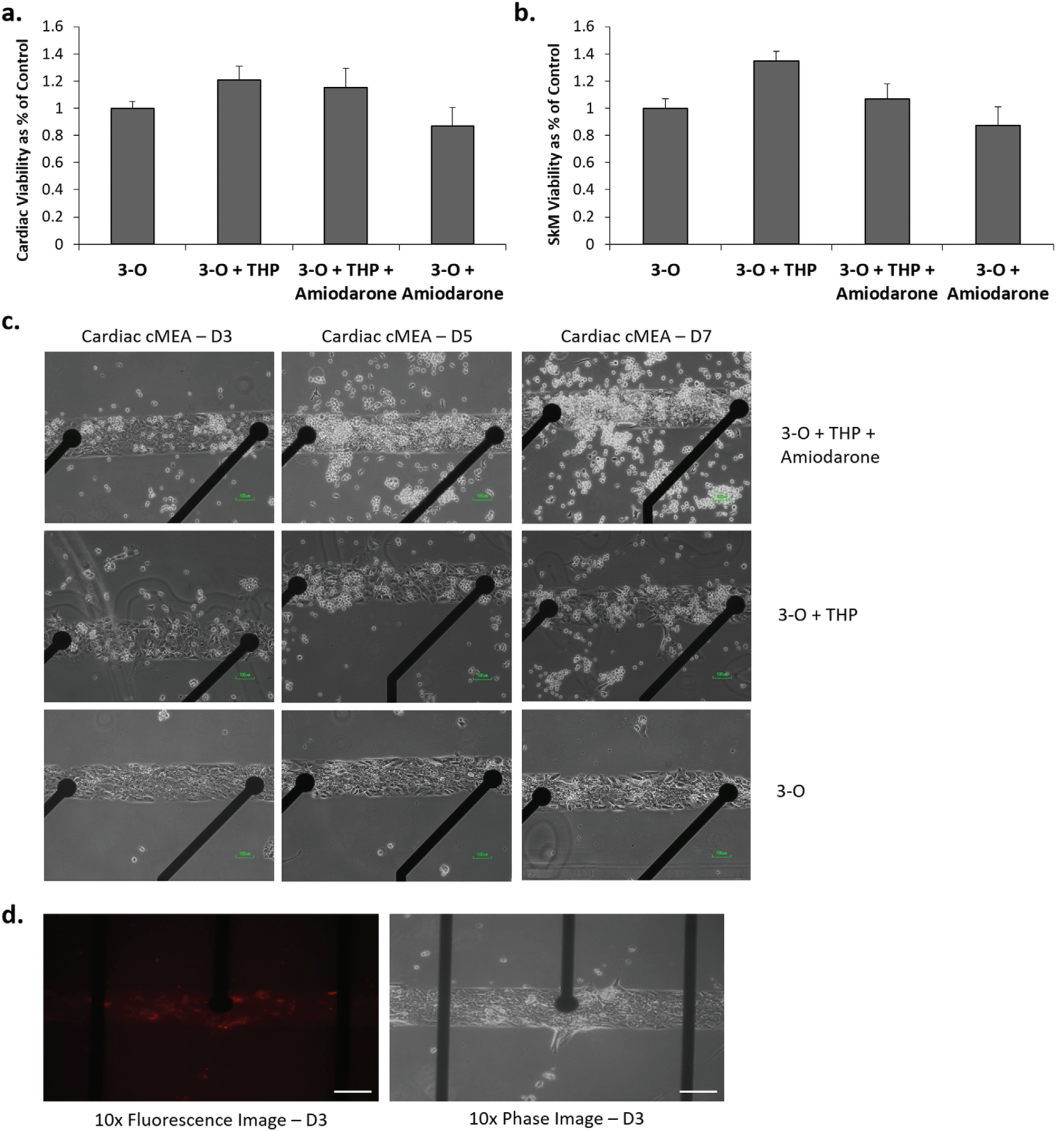
Cardiac and skeletal muscle viability and THP‐1 cell infiltration into amiodarone‐treated 3‐O + THP‐1 systems. a) Cardiac cell viability at day 7, b) skeletal muscle cell viability at day 7, c) phase contrast microscopy images indicating infiltration of THP‐1 cells into the cardiac tissue of amiodarone‐treated systems versus undosed and control systems, and d) fluorescence and phase images of cell tracker loaded THP‐1 cells infiltrated into cardiac MEAs in systems treated with amiodarone. Data points are mean ± SEM. No statistically significant differences observed between conditions in panels (a) and (b).

THP‐1 cell basal activation levels were evaluated by immunocytochemistry on days three and five by flow cytometry on days one, three, and five. These data confirm that THP‐1 cells were not activated as a result of recirculation in the platform, interactions with the three other cell types, or any other intrinsic quality of the system. CD14 and CD16 were used as general markers for monocytes/macrophages and CD69 expression was used as an indicator of activated monocytes.^[^ ^45^ ^]^ Confocal imaging of immunostained THP‐1 cells indicated that CD14 and CD16 expression was high at days 3 and 5, while CD69 expression remained comparatively low (**Figure** **2**a). Further phenotypic analysis of recirculating THP‐1 cells by flow cytometry revealed they maintained high expression of CD14 and CD16 from D1 to D5. Further, ≈10% of these THP‐1 cells were found to be CD69 positive from D1 to D3, with an increase to 20% on D5 (Figure 2b). Datasets for cardiac and skeletal muscle physiology as well as CYP [1A1, 3A4, 2C9] activity and biomarker data for liver physiology were collected and analyzed to set a baseline for comparison to subsequent experiments with the drugs (**Figure** **3**). Previous studies have indicated that these readouts address organ module physiology and are sensitive markers for dysfunction.^[^ ^26^, ^27^ ^]^ Addition of THP‐1 monocytes to the three‐organ systems (3‐O) did not elicit adverse effects on the functionality of the cardiac cantilevers, microelectrode array (MEAs), or skeletal muscle cantilevers (Figure 3). Additionally, the viability of cardiac and skeletal muscle tissues was not significantly altered by monocyte exposure (**Figure** **4**). This 3‐O immune system‐on‐a‐chip served as the control system for subsequent immune cell activation experiments.

### 3.3 Tissue Specific/Tissue Directed Immune Response (Indirect Activation of THP‐1 Cells)

To investigate tissue specific immune response, we treated 3‐O systems ± THP‐1 with 50 × 10^−6^ m amiodarone to determine the responsiveness of THP‐1 cells to selective cardiomyocyte damage. Amiodarone is a well‐characterized cardiotoxic compound affecting beat frequency and force generation.^[^ ^46^ ^]^ Specifically, we determined the level of monocyte activation and infiltration into the tissue chips following acute amiodarone dosing in addition to functional measurements of cardiomyocyte and skeletal muscle function. Our dose–response analysis determined a concentration (50 × 10^−6^ m) that caused physiological dysfunction without inducing cell death (Figure S2, Supporting Information). After amiodarone dosing, medium samples were drawn from the systems 24 h post‐dose and analyzed by HPLC to determine the amount of amiodarone remaining in the systems. A reduction of 97% (50 × 10^−6^ to 1.41 × 10^−6^ m mean concentration) (amiodarone EC_50_: 1.7 × 10^−6^ m) was observed, indicating that nearly all the drug was metabolized by the liver or adsorbed to the housing material after one day (Figure S3, Supporting Information).

The addition of THP‐1 cells to the 3‐O system had no significant effect on cardiac or skeletal muscle physiology (Figure 3). Amiodarone treatment reduced cardiac force output by 75% in 3‐O + THP‐1 systems compared to untreated 3‐O + THP‐1 or 3‐O systems at 2 and 4 days after treatment (Figure 3a). Further, amiodarone treatment reduced cardiac conduction velocity by 20% (Figure 3b) and beat frequency by 45% (Figure 3c) compared to untreated systems. However, a recovery in conduction velocity and beat frequency was observed at day 4 (Figure 3b,c). The presence of THP‐1 cells in the 3‐O + amiodarone systems resulted in a further reduction in cardiac force relative to control 3‐O systems (Figure 3a). Cardiac viability was not decreased as a result of THP‐1 cell addition with or without amiodarone treatment (Figure 4a). As expected, amiodarone had no significant effect on skeletal muscle force generation in 3‐O systems (Figure 3d). Notably, while amiodarone‐treatment of 3‐O + THP‐1 systems resulted in an increase in the metabolic rate of the cardiac cantilever chips due to the presence of THP‐1 cells, there were no changes to skeletal muscle force output with THP‐1 cells present (Figure 3d). This effect was likely caused by the absence of macrophage infiltration into the skeletal muscle modules. These data indicate a damaged‐tissue specific response of the THP‐1 immune cells to the injured cardiac tissue in the 3‐O + THP‐1 system.

To confirm a tissue‐specific response to amiodarone treatment by recirculating THP‐1 cells, systems were monitored for THP‐1 infiltration into the tissue modules during the duration of the experiment. Selective infiltration was evident in phase contrast images taken over the 7‐day course of the experiment (Figure 4c), which indicated THP‐1 cell infiltration into cardiac tissue after amiodarone administration. To confirm THP‐1 cell infiltration into the cardiac tissue, a culture of THP‐1 cells was loaded with CellTracker Red CMTPX dye and added to a 3‐O + THP‐1 system and dosed with amiodarone. After three days, these systems were imaged using epifluorescence microscopy. THP‐1 cells had visibly infiltrated into the cardiac tissue module of amiodarone treated systems (Figure 4d). We did not observe CellTracker labeled THP‐1 cells in liver or skeletal muscle modules in the amiodarone‐only dosed systems.

**Figure 5:**
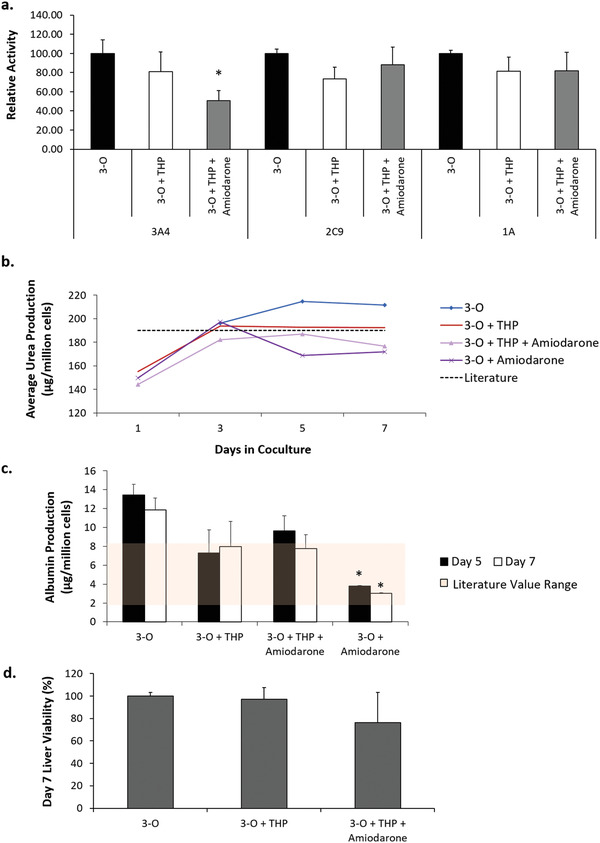
Liver physiology in multiorgan systems treated with amiodarone. a) CYP enzyme activity at day 7 indicating a reduction in CYP3A4 in systems dosed with amiodarone, b) urea production throughout the seven‐day testing period, c) albumin production at days 5 and 7 indicating a reduction for systems treated with amiodarone, beige bar indicates literature values of albumin production, and d) liver viability at day 7. Data points are mean ± SEM.*p ≤ 0.05. No statistically significant differences observed between conditions in panel (d).

Liver physiology was evaluated by endpoint assays at day 7 for the amiodarone treated systems. Specifically, clinically relevant cytochrome p450 enzyme activity was analyzed and quantified. The addition of THP‐1 cells to the systems did not significantly affect CYP3A4, 2C9, or 1A activity (**Figure** **5**a). CYP3A4 activity was found to be reduced in 3‐O + THP‐1 systems that were dosed with amiodarone compared to untreated control systems (Figure 5a), while CYP2C9 and 1A were found to be unchanged by this treatment. Urea production was unaffected by the addition of amiodarone during the time course of the experiment (Figure 5b). However, albumin production was reduced by amiodarone treatment, and this effect was more pronounced in the absence of THP‐1 cells (Figure 5c).

Endpoint MTT or alamar blue viability assays indicated that treatment of systems with amiodarone in the presence or absence of THP‐1 cells did not significantly alter cardiac or skeletal muscle cell viability (Figure 4a,b). Endpoint liver cell viability determination indicated no statistically significant differences in viability in the presence of THP‐1 cells or with the addition of amiodarone or their combination (Figure 5d).

**Figure 6:**
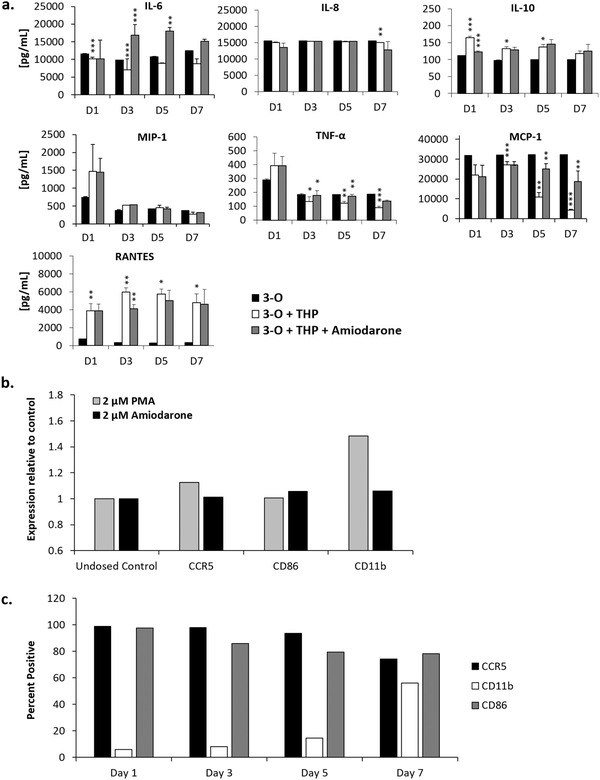
Cytokine release profile and THP‐1 cell activation levels in systems. a) Selected cytokine expression profile throughout analyzed throughout the 7‐day experimental time course, b) flow cytometry quantification of THP‐1 cells expressing CCR5, CD11b, and CD86 pre‐ and post‐direct PMA, or amiodarone treatment, and c) flow cytometry quantification of THP‐1 cells removed from 3‐O + THP‐1 systems expressing CCR5, CD11b, and CD86 pre‐ and post‐amiodarone treatment. Data points are mean ± SEM, *p ≤ 0.05, **p ≤ 0.01, ***p ≤ 0.001.

Release of cytokines IL‐6, IL‐8, IL‐10, MIP‐1, TNF‐*α*, MCP‐1, and RANTES were tracked throughout the time course of the experiment. IL‐10, TNF‐*α*, RANTES and MIP‐1 concentrations were increased on day 1 by the addition of THP‐1 cells compared to 3‐O systems, while addition of amiodarone increased levels of IL‐6 and TNF‐*α* relative to the 3‐O + THP control (**Figure** **6**a). The elevated RANTES concentration was as direct result of THP‐1 cell addition and was not notably influenced by amiodarone addition. Additionally, elevated IL‐6 medium concentration in amiodarone‐dosed systems compared to undosed systems suggests an active role for the cytokine in amiodarone‐mediated cardiotoxicity repair. Importantly, the concentrations of the elevated cytokines TNF‐*α*, MIP‐1 and IL‐6 began to resolve during the experiments indicating an immune‐response‐to‐injury phenotype.

THP‐1 cell activation levels were monitored by flow cytometry during the amiodarone treatment. A positive control for activation was performed by dosing a culture of THP‐1 cells with 2 × 10^−6^ m phorbol myristate acetate (PMA), which has been shown to drive the differentiation of THP‐1 monocytes into macrophages (Figure 6b).^[^ ^47^ ^]^ This differentiation was evidenced by an 12% and 48% increase in CCR5 and CD11b expression respectively, both of which are commonly used as markers for monocyte differentiation.^[^ ^48^, ^49^ ^]^ C86, a marker for macrophage proinflammatory activation, remained relatively unchanged (Figure 6b). Amiodarone treatment of THP‐1 cells alone did not significantly alter their viability or levels of CCR5, CD86, or CD11b expression (Figure 6b).

Taken together these data indicate that amiodarone does not directly activate THP‐1 macrophages and that THP‐1 cell activation was caused by the damaged cardiomyocytes and was indicative of a M2 phenotype. Recirculating THP‐1 cells were removed from treated 3‐O + THP + Amiodarone systems on days 1, 3, 5, 7 and analyzed by flow cytometry for their expression of CCR5, CD11b, and CD86 receptors. Expression of CCR5 and CD86 remained relatively stable over the six‐day window, while CD11b increased. These data indicate that CD11b is an important indicator of THP‐1 cell activation in response to amiodarone treatment and subsequent cardiac cell damage (Figure 6c). Additionally, a direct comparison of CCR5, CD86, and CD11b expression by THP‐1 cells removed from 3‐O + THP and 3‐O + THP + Amiodarone systems indicated that amiodarone treatment of 3‐O + THP systems increased expression of CCR5 and CD86 by THP‐1 cells in these systems, suggesting an inflammatory response by the recirculating monocytes^[^ ^50^ ^]^ (Figure S4, Supporting Information).

### 3.4 Nonspecific Activation of Immune Cells in the Three‐Organ Platform

The immune system also can be systemically activated and recapitulating this state is a vital function necessary for an immune system‐on‐a‐chip assay platform. To evaluate this capability, compounds known to activate recirculating monocytes and promote organ infiltration and nonselective organ system damage, THP‐1 cells in systems were treated with LPS and IFN‐*γ*. These compounds were selected due to the robust effects they elicit on nonselective monocyte differentiation and activation. A cocktail of LPS (10 ng mL^−1^) and IFN‐*γ* (100 U mL^−1^) was added to the 3‐O systems on day 3 after assembly.

**Figure 7:**
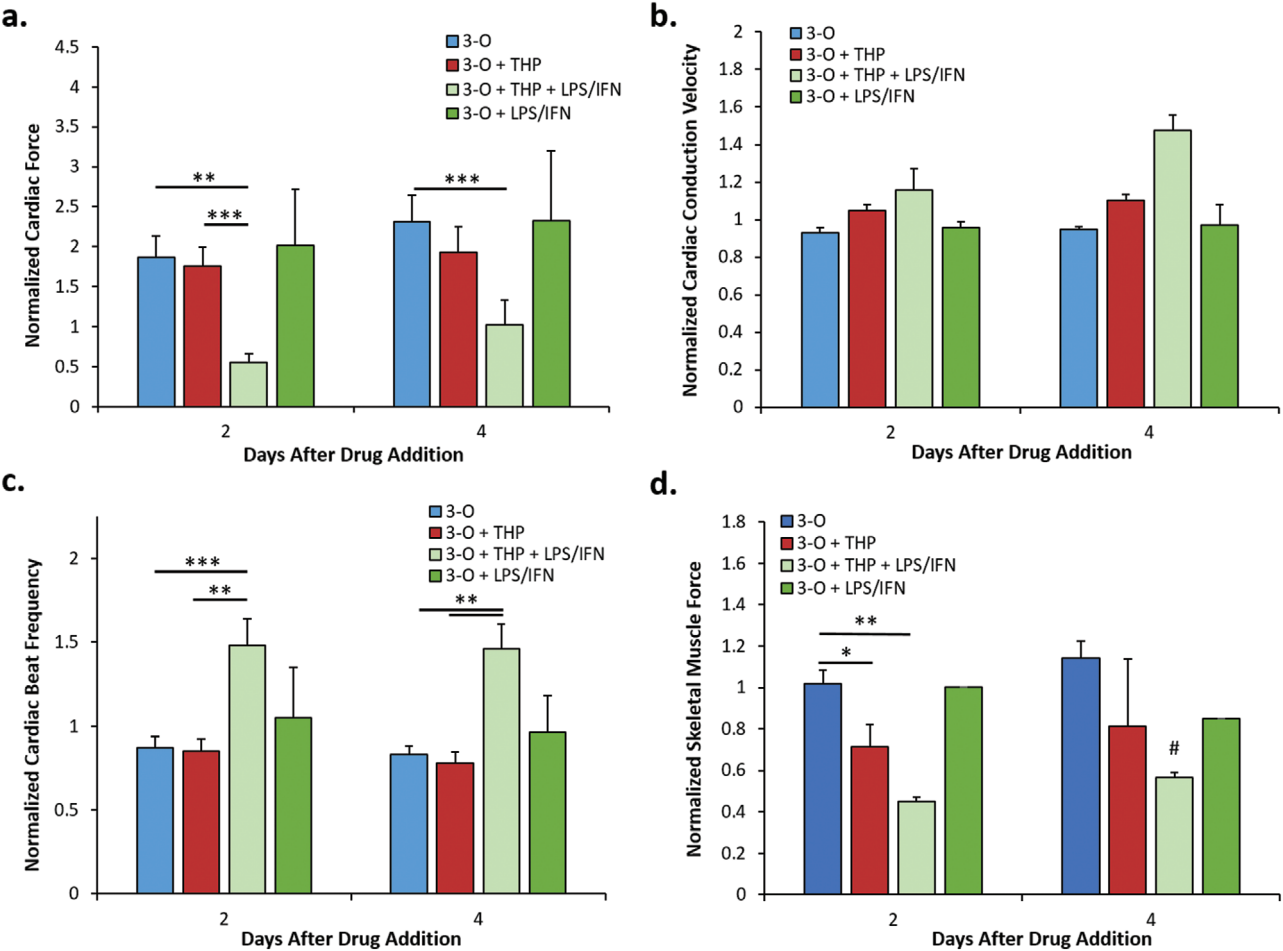
Functional evaluation of cardiomyocytes and skeletal muscle myotubes in LPS and IFN‐*γ* treated 3‐O + THP‐1 systems. a) Normalized cardiac force measurements from cantilevers, b) normalized cardiac conduction velocity measurements from MEAs, c) normalized beat frequency measurements from cantilevers, and d) normalized skeletal muscle force measurements from cantilevers. Data points are mean ± SEM, *p = 0.05, **p ≤ 0.01, ***p ≤ 0.001, #*p* = 0.07. No statistically significant differences observed between conditions in panel (b).

LPS/IFN‐*γ* addition in the absence of THP‐1 had no statistically significant effect on cardiac force, cardiac conduction velocity, or cardiac spontaneous beat frequency (**Figure** **7**). On day 2, skeletal muscle force was reduced 25% by the addition of THP‐1 cells and a further 30% by the addition of LPS/IFN‐*γ* (Figure 7d). On day 4, skeletal muscle force increased 10% in the THP + LPS/IFN‐*γ* condition compared to day 2 (*p* = 0.07) (Figure 7d).

3‐O + THP‐1 systems treated with LPS/IFN‐*γ* exhibited a 70% reduction in cardiac contractile force, a 30% increase in cardiac conduction velocity on day 4, and a 40% reduction in skeletal muscle force compared to control 3‐O + THP‐1 systems (Figure 7a,b). Cardiac spontaneous beat frequency in 3‐O + THP‐1 systems increased 60% by the addition of the LPS/IFN‐*γ* cocktail (Figure 7c). These data, indicating system‐wide changes to cardiac and skeletal muscle physiology, were different due to the differential nature of THP‐1 cell activation.

**Figure 8:**
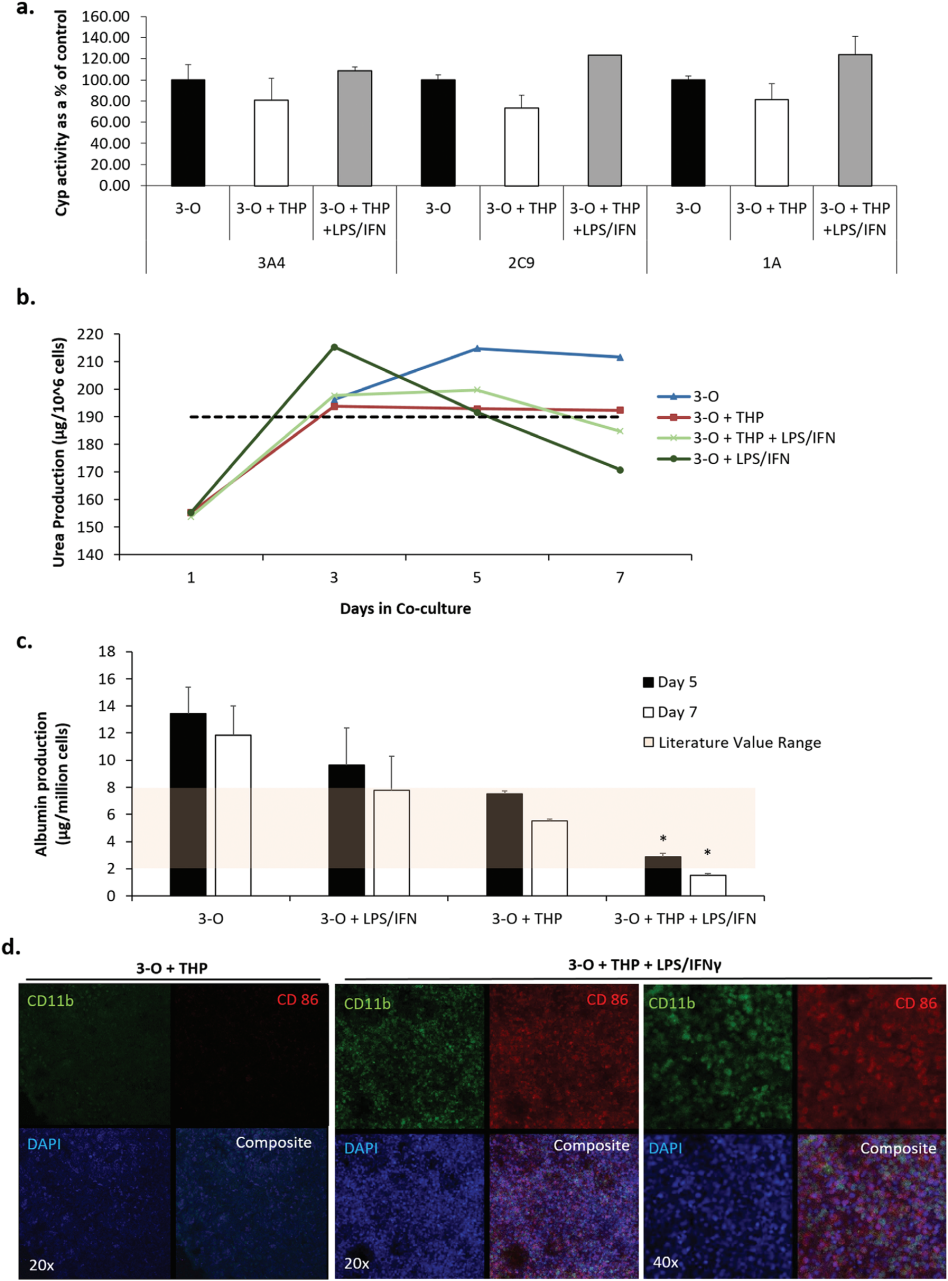
Functional evaluation of liver physiology in LPS and IFN‐*γ* treated 3‐O + THP‐1 systems. a) Normalized, day 7 Cyp 3A4, 2C9, and 1A activity, b) urea production over seven days, c) albumin production at days 5 and 7, beige bar indicates literature values of albumin production, and d) immunocytochemical evaluation of THP‐1 infiltration into liver in undosed 3‐O + THP‐1 and LPS/IFN‐*γ*‐dosed 3‐O + THP‐1 systems indicating increased THP‐1 cell infiltration after dosing. Data points are mean ± SEM. *p ≤ 0.05. No statistically significant differences observed between conditions in panel (a).

Cyp3A4, 2C9, and 1A activity in the liver were evaluated at day 7. LPS/IFN‐*γ* addition had no effect on Cyp3A4 activity, but elevated 2C9 and 1A activity compared to standard activity in untreated 3‐O + THP‐1 systems (**Figure** **8**a). Hepatocyte physiology was also evaluated by measuring urea and albumin production. Urea production was 182.8 ± 4.3 µg per million cells and was not statistically different in any of the treatment conditions with or without THP‐1 cell addition (Figure 8b). The addition of LPS/IFN‐*γ* did not statistically affect albumin production. Albumin production in 3‐O + THP‐1 cells was 6.8 ± 1.1 µg per million cells. However, the addition of LPS/IFN‐*γ* to the 3‐O + THP‐1 cell system reduced albumin production at days 5 and 7 compared to undosed 3‐O + THP‐1 cell systems (Figure 8c). Immune cell infiltration was evident in the liver module by phase microscopy, and this infiltration was confirmed by immunocytochemistry (Figure 8d, left panel). CD11b staining in the liver tissue module was increased in the LPS/IFN‐*γ* treated systems compared to the controls, indicating THP‐1 cell infiltration (Figure 8d, middle and right panels)

**Figure 9:**
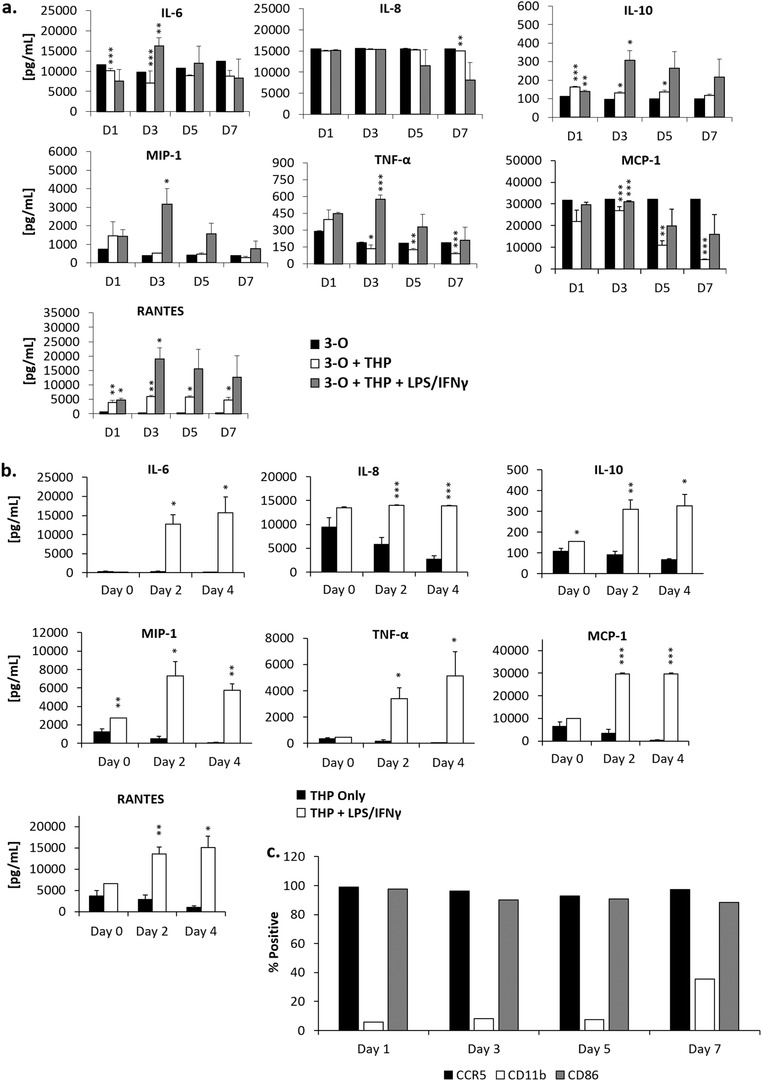
LPS + IFN‐*γ* induced cytokine release profile and THP‐1 cell activation status. a) Selected cytokine expression profile analyzed throughout the 7‐day experimental time course, b) temporal cytokine release from THP‐1 cells in housings following LPS + IFN*γ* treatment, c) THP‐1 activation status in 3‐organ systems before and after LPS + IFN‐*γ* treatment. Data points are mean ± SEM, *p ≤ 0.05, **p ≤ 0.01, ***p ≤ 0.001.

Release of cytokines IL‐6, IL‐8, IL‐10, MIP‐1, TNF‐*α*, MCP‐1, and RANTES was quantified on days 1, 3, 5, and 7. LPS/IFN‐*γ* addition was shown to significantly increase concentrations of IL‐6, IL‐10, MIP‐1, TNF‐*α*, MCP‐1, and RANTES compared to untreated 3‐O + THP systems (**Figure** **9**a). Concentrations of these cytokines were also elevated in LPS/IFN‐*γ* dosed systems that only contained recirculating THP‐1 cells, indicating that THP‐1 cell activation was primarily induced by the LPS/IFN‐*γ* activation cocktail (Figure 9b). THP‐1 CCR5, CD11b, and CD86 receptor expression was evaluated at day 1, 3, 5, and 7 of recirculation with the LPS/IFN‐*γ* being added on day 3 after THP‐1 cells were drawn from the systems. No significant changes in receptor expression were observed until day 7, when CD11b expression was elevated 7‐fold (Figure 9c).

## 4 Discussion

We have established a microfluidic, human‐on‐a‐chip system with a functional immune cell component utilizing a monocyte‐derived cell line (THP‐1) and demonstrated selective and nonselective monocyte/macrophage activation in the presence of three functional tissues. More importantly, we have observed a selective cytokine release profile that is specific for each condition. The introduction of a recirculating immune component in a human‐on‐a‐chip platform allows this system to monitor complex cell behavior and tissue–immune cell interactions in response to drug treatment. One advantage of this microfluidic system is the ability to analyze immune responses in a controlled, serum‐free environment.^[^ ^51^ ^]^ THP‐1 monocytes have been extensively studied in basic and translation research and are well‐suited to study immune responses while the cells are monocytes, and as macrophages following differentiation.^[^ ^52^, ^53^, ^54^ ^]^ THP‐1 cells differentiate into macrophages following stimulation with LPS and subsequently express inflammatory cytokines.^[^ ^52^ ^]^ When compared to primary human peripheral blood monocytes, THP‐1 cells have been shown to be less responsive to LPS, express reduced levels of CD14 and produce less IL‐8.^[^ ^22^ ^]^ However, these studies were conducted using cells in monoculture, under static conditions, and in the presence of fetal bovine serum (FBS); all factors known to influence cell physiology. Another advantage of this multiorgan, microphysiological system is that it more accurately mimics in vivo conditions when compared to static monocultures (e.g., circulation, cell–cell interactions, and defined medium conditions).

In this report, we described the effects of two unique treatment protocols in a 3‐O system containing recirculating THP‐1 monocytes. The treatments were intended to drive THP‐1 cell activation toward either a classical nonselective M1 phenotype or the alternative M2 tissue repair phenotype to study the effects these different pathways have on the other tissues in the system and their downstream interactions with each other.

Initially, the system was treated with amiodarone, a compound known to have cardiotoxic effects at micromolar concentrations in vitro. We conducted a dose–response curve for cardiotoxicity in the platform and chose the concentration that damaged but did not kill the cells (50 × 10^−6^ m). This allowed the study of functional cardiotoxicity, and the cytokines produced by the damaged cardiomyocytes, without the confounding variability of the release of necrotic debris recirculating in the system (Figure S2, Supporting Information). As indicated by the functional readouts in Figure 3, amiodarone treatment impaired cardiac physiology while sparing skeletal muscle function two days post‐treatment and resulted in selective THP‐1 cell infiltration into the cardiac tissue chips (Figure 4d). These findings are similar to previous reports indicating reduced beat frequency following amiodarone treatment.^[^ ^55^, ^56^ ^]^ Additionally, these data support literature observations that cytokines, particularly TNF‐*α*, can negatively affect cardiac physiology.^[^ ^57^, ^58^ ^]^ Interestingly, four days post‐treatment a recovery of function in conduction velocity and beat frequency was observed, but not in cardiac force. This functional recovery coincided with a resolution of the cytokine elevation observed immediately following treatment with amiodarone. Generally, these data are supported by findings indicating that nonresident, infiltrating macrophages are important for modulating the wound remodeling phenotype in the adult heart including short‐term fibrosis and long‐term repair.^[^ ^59^ ^]^ As expected, liver CYP3A4 activity was decreased by amiodarone treatment while CYP2C9 and CYP1A were not affected (Figure 5). THP‐1 cell activation levels were assessed by flow cytometry throughout the duration of the experiment. Amiodarone had no direct effect on recirculating THP‐1 cell activation, however, when systems containing recirculating THP‐1 cells were dosed with amiodarone there was a delayed, but pronounced, induction of CD11b suggesting cytokines produced by damaged cardiomyocytes initiated THP‐1 activation (Figure 6b,c). These data corroborate findings by multiple groups that show an important role for macrophages in cardiac remodeling post‐myocardial infarction in vivo and in vitro.^[^ ^60^, ^61^, ^62^ ^]^ However, none of the in vitro studies were in multiorgan recirculating systems. From the cytokine data the exact program driving THP‐1 cell activation is unclear, however, the increasing and sustained levels of IL‐6 from day 3 through day 7 suggests it played a role in the increased expression of CD11b on THP‐1 macrophages (Figure 6a) and may be a useful marker for cardiac‐damage driven activation. Further, the resolution of elevated levels of RANTES and IL‐10 suggest a repair phenotype for the THP‐1 cells. The resolution of the IL‐10 levels after treatment was similar to the observations of Groger et al.^[^ ^21^ ^]^ Overall, these data indicate that targeted tissue damage in this HoaC system resulted in selective infiltration of THP‐1 monocytes to the site of damage and a specific cytokine biomarker “fingerprint” in the form of elevated IL‐6 concentration at days 5 and 7, indicative of differentiation into an M2 macrophage phenotype.

Systems were also treated with LPS and IFN‐*γ*, known to drive robust nonselective monocyte activation and acute inflammation in vivo and in vitro. This treatment drives a proinflammatory, cytokine‐storm/sepsis‐like scenario and facilitates an investigation into the effects of “classical” monocyte activation to an M1 macrophage phenotype that affected cardiac, skeletal muscle, and liver function. This conclusion is supported by the continued elevation of cytokines in the system compared to the amiodarone‐dosed systems. Furthermore, when compared to amiodarone treated systems, the divergent approach to macrophage activation and the unique cell responses facilitate the investigation into monocyte immune responses to unknown compounds. Specifically, does a potential therapeutic candidate cause indirect, tissue‐driven immune cell responses, or does it directly activate circulating monocytes, which could be especially useful for evaluating biologics, specifically antibodies which can cause acute anaphylaxis, serum sickness, and a host immune response to antibody treatment.^[^ ^63^, ^64^ ^]^

As observed with amiodarone treatment of THP‐containing systems, cardiac physiology was impaired when THP‐containing systems were treated with LPS + IFN‐*γ* (Figure 7a–c). These data indicate a difference in THP‐1 cell response to different activation programs, one targeted and the other holistic. These data suggest that continuous elevation of IL‐6 concentration in the medium while other cytokine concentrations return to baseline could be a useful biomarker for compounds that damage specific tissues. Specifically, this observation suggests that IL‐6 elevation in the medium may also serve a diagnostic role in addition to its well‐established role as a prognostic indicator of myocardial infarction and heart failure.^[^ ^65^, ^66^ ^]^ Moreover, the absence of elevated IL‐6 medium concentrations at days 5 and 7 in the LPS/IFN‐*γ*‐treated systems support a role for this cytokine in targeted, tissue‐specific compound damage. The elevated levels of TNF‐*α* immediately after treatment followed by declining levels after treatment confirms findings by Groger et al. in their liver + THP‐1 microphysiological system.^[^ ^21^ ^]^

Liver physiology was also assessed during LPS + IFN‐*γ* treatment of THP‐1 containing systems. As neither LPS nor IFN‐*γ* are metabolized by liver CYP enzymes, no statistically significant functional deficits were observed in CYP activity when the experiment concluded at day seven (Figure 8a). Furthermore, urea production remained stable throughout the duration of the experiment (Figure 8b). While the CYP and urea data suggest a healthy liver module in the presence of recirculating THP‐1 cells treated with LPS + IFN‐*γ*, albumin production was significantly decreased in these conditions compared to systems treated with LPS + IFN‐*γ* alone or with recirculating THP‐1 cells alone (Figure 8c). The observation of hypoalbuminemia in the context of monocyte‐mediated inflammation confirm previous human and rodent studies investigating inflammation‐induced reductions in liver synthesis.^[^ ^67^, ^68^ ^]^ Taken together, these data indicate liver albumin production's sensitivity to compound treatment and THP‐1 cell infiltration. In the absence of any single biomarker of liver function, the stratification of the results makes these an ideal group of tests for assessing liver function in presence of compounds with unknown effects.^[^ ^69^ ^]^ Further experimentation will be necessary to reveal the mechanisms involved in the sensitivity of albumin production in these conditions.

## 5 Conclusion

Here, we demonstrate a multiorgan system human‐on‐a‐chip device containing a functional immune component. This platform supports the interrogation of complex, integrated immune system responses to tissue‐level effects caused by drug treatment. We demonstrated a differential THP‐1 cell response to compounds that indirectly or directly activated monocytes and drove macrophage differentiation. These findings support the idea that HoaC systems can be used to determine broad differences in immune cell activation. This is especially important as the in vitro tools for deciphering how immune cells interact with parenchymal cells to mediate immune responses to inflammation are limited.^[^ ^69^ ^]^ This system's engineering parameters, primarily its gravity‐driven serum‐free medium recirculation and reconfigurable nature, are ideal for the integration and analysis of other immune components either in isolation or in combination by including PBMCs and T‐cells. For example, integration of a lymphatic tissue chip would enable analysis of innate activation of adaptive immune responses including lymphocyte migration, activation, and expansion. Additionally, the effects of inflammatory mediators on vessel permeability and leukocyte extravasation could be investigated by combining immune cells with a microphysiological blood and lymphatic vessel system.^[^ ^70^ ^]^ The bio‐MEMS chips facilitate noninvasive testing of functional parameters and the low system volume supports analysis of cytokines and other molecules produced in small amounts by tissues in the system. Taken together, we believe this system provides an ideal platform to study innate and adaptive as well as cell‐based and humoral immune responses in a self‐contained multiorgan system.

## Author Contributions

T.S. and J.W.R. contributed equally to this work. T.S., J.W.R., L.R.B., and D.E. conducted experiments; T.S., J.W.R., C.W.M., C.J.L., F.S., A.R., C.B.‐L., M.L.S., J.J.H. developed experimental design; T.S., J.W.R., C.W.M., L.R.B., C.J.L., F.S., A.R., C.B.‐L., M.L.S., and J.J.H. performed the data analysis; T.S. and C.J.L. calculated the statistics; T.S., J.W.R., C.W.M., L.R.B., M.L.S., and J.J.H. wrote the manuscript.

## Conflict of Interest

The authors confirm that competing financial interests exist but the financial support for this research did not influence its outcome.

## Supporting information

Supporting Information

Supplemental Video 1

Supplemental Video 2

Supplemental Video 3

Supplemental Video 4
