# Supplementary material for: Differential Monocyte Actuation in a Three‐Organ Functional Innate Immune System‐on‐a‐Chip
Source: Adv Sci (Weinh). 2020 Jun 2;7(13):2000323. doi: 10.1002/advs.202000323 (PMC7341107; doi:10.1002/advs.202000323)
Supplement: Supplementary file 1 — Supporting Information [file ADVS-7-2000323-s001.pdf]

Differential Monocyte Actuation in a Three-Organ Functional Immune System-on-a-Chip

Authors: Trevor Sasserath[1*], John W. Rumsey[1*], Christopher W. McAleer[1], L. Richard Bridges[1], Christopher Long[1], Daniel Elbrecht[1], Franz Schuler[2], Adrian Roth[2], Christina Bertinetti-LaPatki[2], Michael L. Shuler[1], James J. Hickman[1,&]

**Supplemental Materials**

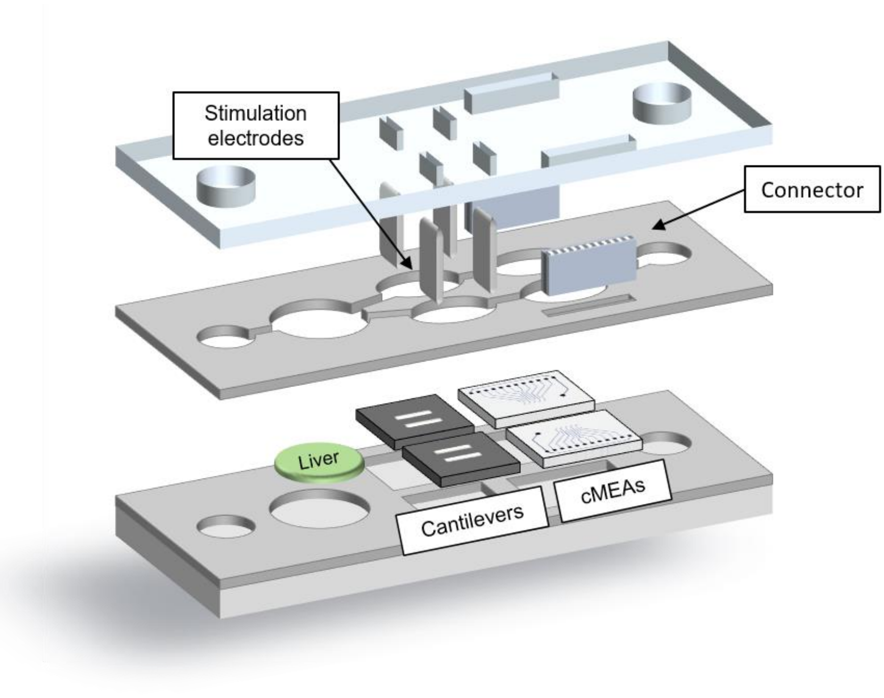


**Supplemental Figure 1. Diagram of the 3-organ+THP-1 cell immune-on-a-chip system.** The lower housing (bottom) and gasket hold the liver coverslip, the cantilever arrays, and the cMEAs. The upper gasket (middle) defines the recirculating medium and THP-1 cells flow path. The upper housing (top) contains the medium change access ports and slots for the zebra connectors.

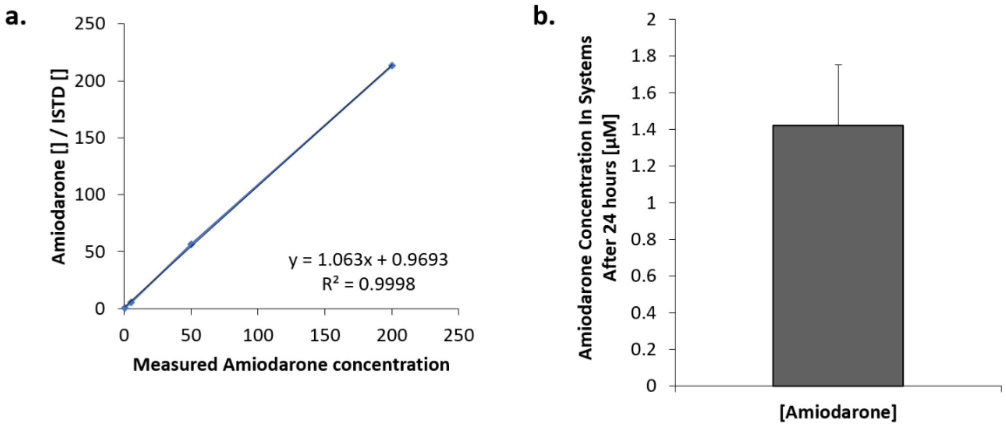


**Supplemental Figure 2**. Amiodarone Concentration in 3-O+THP-1 Systems. (a) HPLC determined amiodarone concentration vs. standard curve, (b) amiodarone concentration in 3-O+THP-1 systems 24-hours after compound addition.

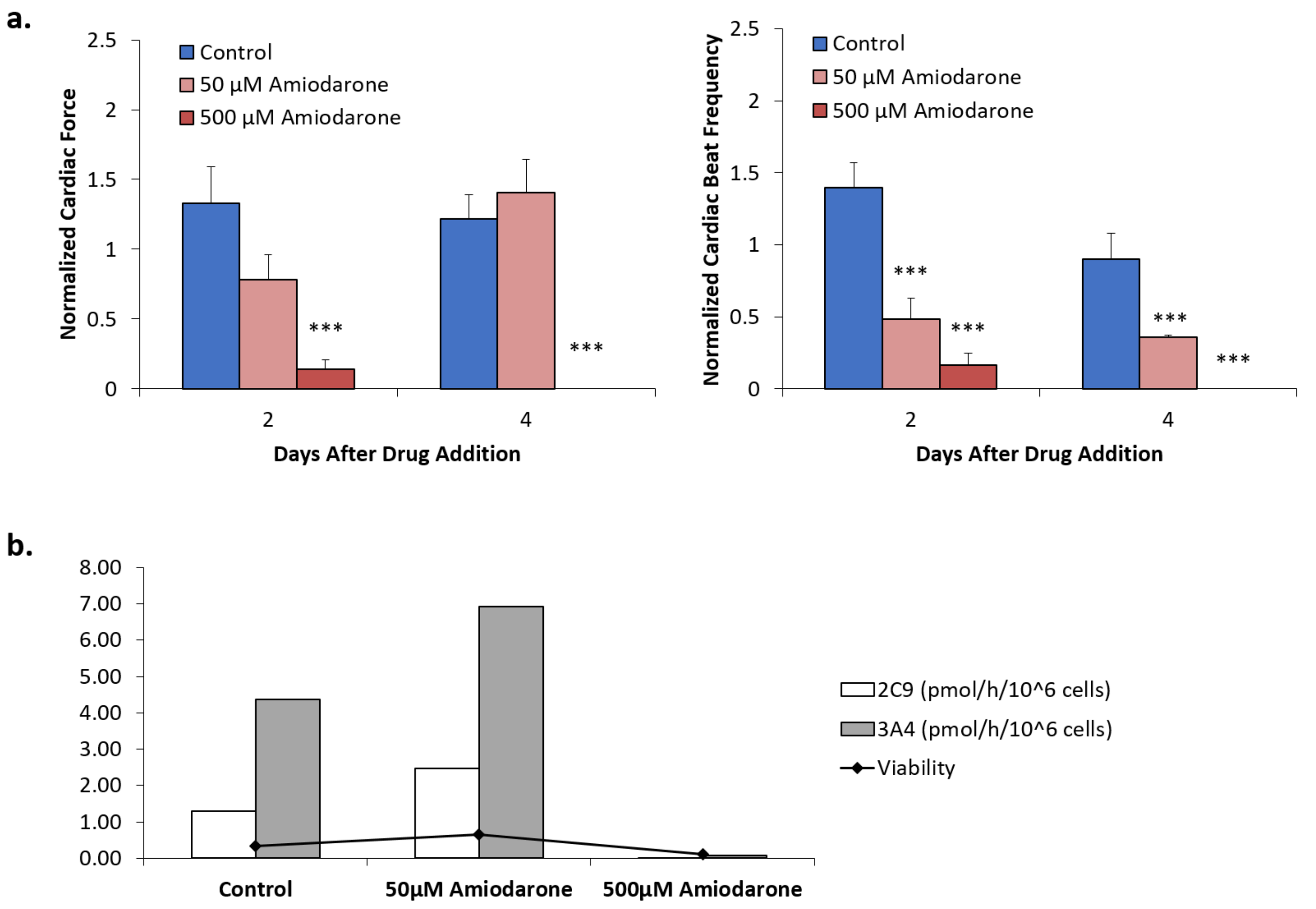


**Supplemental Figure 3**. Effects of Amiodarone Dose Response on Cardiac and Liver Function in 3-O Systems. (a) Normalized cardiac force and cardiac beat frequency from cantilevers, (b) hepatocyte viability and relative activities of CYPs 2C9 and 3A4 four days post-dose. Data points are mean ± SEM. *** $p \leq 0.001$. Day 4 column for 500 µM amiodarone denotes cessation of activity.

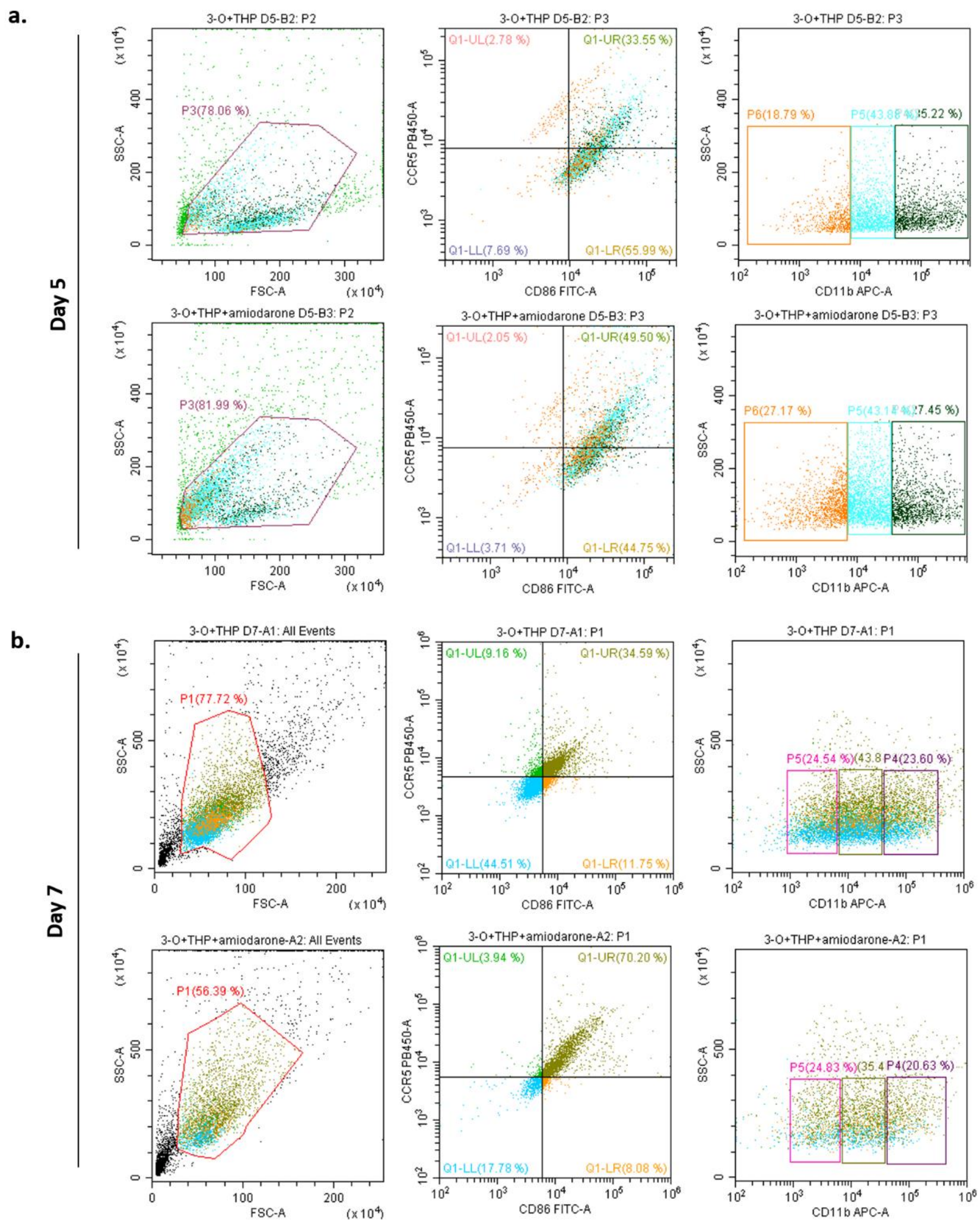


**Supplemental Figure 4.** Amiodarone treatment of 3-O+THP systems increases expression of CCR5 and CD86. (a) Scatter plot showing expression of CCR5, CD86, and CD11b by THP-1 cells removed from 3-O+THP and 3-O+THP+Amiodarone systems on day 5 and (b) day 7. Lower granularity of THP-1 cells from undosed systems indicates a greater population of inactivated cells compared to THP-1 cell from amiodarone-dosed systems.

**Supplementary Videos**

Video 1: Day 1 Recirculating THP cells in systems

Video 2: Day 3 Recirculating THP cells in systems

Video 3: Day 5 Recirculating THP cells in systems

Video 4: Day 7 Recirculating THP cells in systems
